# Key Mechanisms of Oxidative Stress-Induced Ferroptosis in Heart Failure with Preserved Ejection Fraction and Potential Therapeutic Approaches

**DOI:** 10.31083/RCM26613

**Published:** 2025-03-25

**Authors:** Junling Lin, Bingtao Li, Xueqi Guo, Guodong Li, Qi Zhang, Wenjuan Wang

**Affiliations:** ^1^Department of Cardiovascular Center, First Affiliated Hospital of Huzhou University, 313000 Huzhou, Zhejiang, China

**Keywords:** oxidative stress, ferroptosis, preserved ejection fraction heart failure, targeted anti-cancer drugs, apatinib

## Abstract

The prevalence of heart failure with preserved ejection fraction (HFpEF) is increasing annually, particularly among patients with metabolic disorders such as hypertension and diabetes. However, there is currently no treatment capable of altering the natural course of HFpEF. Recently, the interplay between oxidative stress and ferroptosis in cardiovascular diseases has drawn extensive attention; however, minimal research has been published on the mechanisms of oxidative stress and ferroptosis in HFpEF. This paper reviews the relevant mechanisms through which oxidative stress is induced and promotes ferroptosis during the development of HFpEF. The review also explores more efficacious treatment approaches for HFpEF by inhibiting oxidative stress and ferroptosis, thereby offering a theoretical foundation for verifying the feasibility of these methods for further research. As tumor-targeted therapy progresses, the survival period of tumor patients is prolonged, and cardiovascular events have gradually emerged as one of the most crucial causes of death among tumor patients. Hence, inhibiting the vascular endothelial growth factor (VEGF) pathway has become a major target in tumor treatment, significantly enhancing patient survival. Nevertheless, secondary cardiovascular complications and events, such as myocardial injury and subsequent heart failure, have severely impacted patient survival and quality of life. Therefore we have also explored the potential mechanism through which novel targeted anti-cancer drugs induce HFpEF via ferroptosis. Additionally, we reviewed the specific modes of action for preventing and treating HFpEF without influencing their anti-cancer therapeutic effect.

## 1. Introduction

Heart failure (HF) constitutes a clinical state at the advanced or end stage of diverse cardiovascular disorders, encompassing hypertension, coronary artery atherosclerotic heart disease, and arrhythmias. Currently, the prevalence of HF among adults worldwide ranges from 1% to 3%, with the prevalence rate in China currently at 1.1%. The incidence of this disease rises markedly with age, posing a severe threat to human life and health [1]. Heart failure with preserved ejection fraction (HFpEF) constitutes one of the principal types of HF. The most frequent cause of HFpEF is hypertension, which accounts for 80% of HFpEF [2]. The pathophysiological mechanisms of HFpEF encompass multiple biological systems. In addition to mitochondrial autophagy, inflammatory responses, and oxidative stress, oxidative stress also assumes an important role in HFpEF. Oxidative stress plays a significant role in the occurrence and deterioration of HFpEF by upregulating ferroptosis. The issue of cardiovascular damage related to novel targeted anti-cancer therapeutics has attracted increasing attention. Vascular endothelial growth factor (VEGF) inhibitors induce cardiovascular toxicity mainly in the form of hypertension and cardiac dysfunction, which might eventually result in the development of HFpEF. During this process, oxidative stress and the upregulation of ferroptosis pathways play crucial roles in the pathogenesis. Consequently, an in-depth exploration of the mechanism through which oxidative stress leads to the upregulation of the ferroptosis pathway and its association with HFpEF is of paramount importance for clinical diagnosis and treatment.

## 2. The Prevalence and Associated Mechanisms of Heart Failure with Preserved Ejection Fraction

Heart failure can be classified into four fundamental types according to left ventricular ejection fraction (LVEF): heart failure with reduced ejection fraction (HFrEF), heart failure with mildly reduced ejection fraction (HFmrEF), heart failure with improved ejection fraction (HFimpEF), and HFpEF [3]. Among them, HFpEF is increasing at a rate of 1% annually and is gradually evolving into one of the most prevalent types of heart failure. The incidence of HFpEF ranges from 1.1% to 5.5%, accounting for 40% to 71% of all heart failure patients [4]. Consequently, greater attention and priority needs to be devoted to exploring the pathogenesis and treatment of HFpEF.

The pathogenesis of HFpEF is heterogeneous, encompassing the activation of the sympathetic nervous system and the renin-angiotensin-aldosterone system (RAAS system), endothelial dysfunction, myocardial fibrosis, inflammation, and mitochondrial autophagy [4, 5]. Recent research has indicated that oxidative stress-induced ferroptosis plays a vital role in the occurrence and development of HFpEF. Oxidative stress-induced ferroptosis can lead to abnormal electrophysiological activity of the cardiac muscle and mitochondrial damage, which has been implicated in the development of HFpEF [6, 7]. Iron-dependent cell death, referred to as ferroptosis, is recognized as a potential key factor in cardiovascular diseases [8]. A recent study found that inhibiting ferroptosis has been demonstrated to reverse HFpEF [9].

The widespread utilization of anti-cancer drugs has enhanced the survival rate of cancer patients; however, it has concurrently brought about severe cardiac toxicity. For instance, anthracycline antineoplastic drugs, which have been extensively employed in the treatment of various malignant tumors [10, 11], also induce cardiac toxicity involving ferroptosis. A study has revealed that the inhibition of ferroptosis can be utilized to ameliorate the cardiac damage caused by doxorubicin [12]. Targeted anti-cancer small molecule tyrosine kinase inhibitors (TKIs), such as Apatinib, are capable of exerting selective inhibitory effects on the VEGF and specific kinases in the body [13]. The cardiotoxicity induced by TKI is classified as “on-target” and “off-target” effects. The former represents the consequence of the drug’s direct action on the cardiac target, whereas the latter constitutes the adaptive and adverse responses of cardiac cells to the pharmacological action of the drug [13, 14]. In our previous study, we discovered that oxidative stress is involved in the cardiovascular damage induced by VEGF. When Apalutamide was employed as an intervention, it significantly enhanced inducible nitric oxide synthase (iNOS) and reduced endothelial nitric oxide synthase (eNOS) levels, resulting in a further decline in nitric oxide (NO) levels, which has emerged as an important mechanism for developing hypertension [15]. Hypertension can induce myocardial injury, further contributing to the development of HFpEF. This process might also encompass the regulation of oxidative stress and ferroptosis. Hence, it is of vital importance to explore the pathogenic mechanisms related to oxidative stress and ferroptosis in HFpEF-related disorders and develop new targets for future therapeutics.

## 3. The Function of Oxidative Stress in HFpEF

**Fig. 1.**
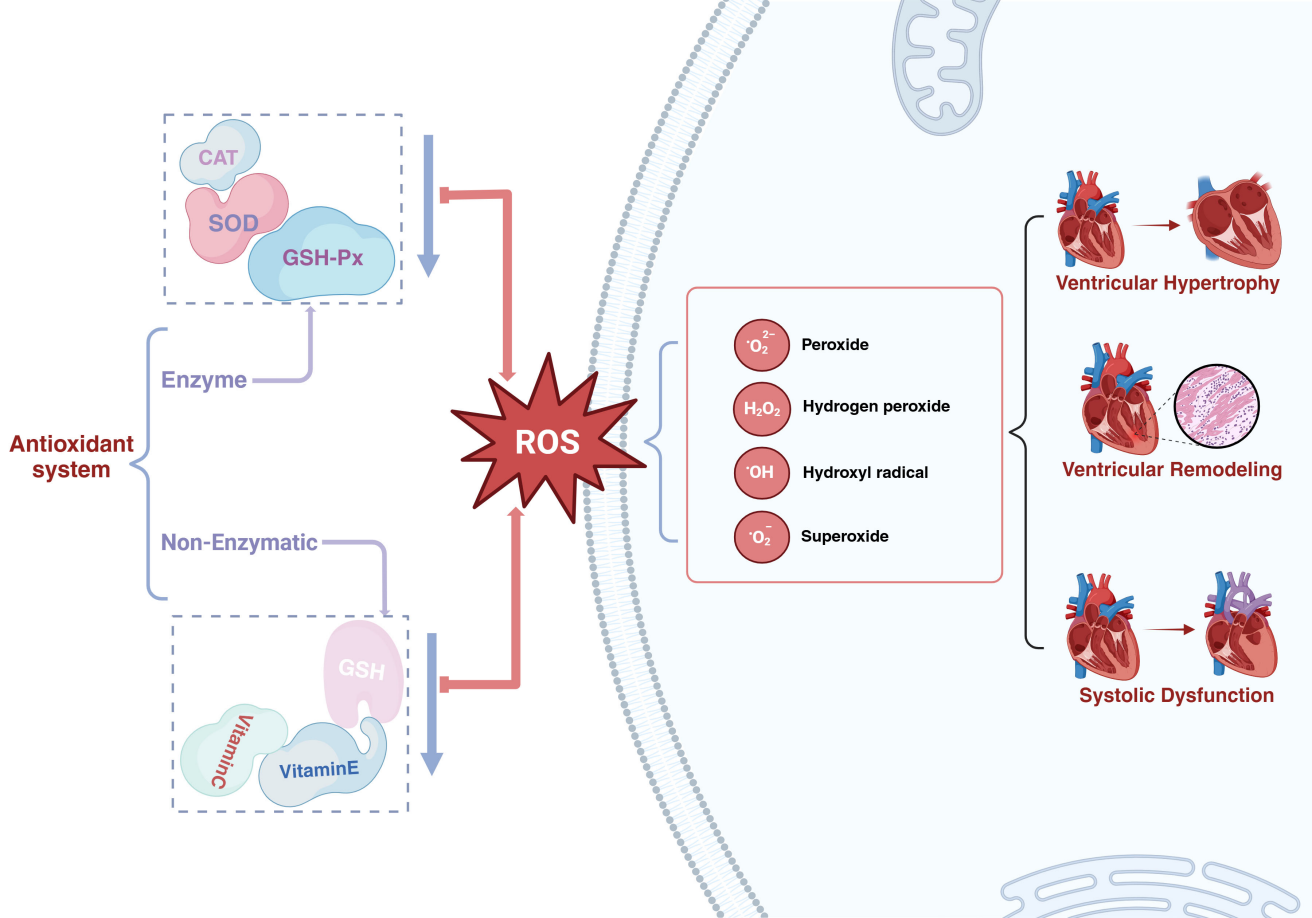
**The function of oxidative stress in heart failure with preserved ejection fraction**. SOD, superoxide dismutase; GSH-Px, glutathione peroxidase; CAT, catalase; GSH, glutathione; ROS, reactive oxygen species. Created in BioRender.com.

Oxidative stress (OS) is defined as a state of imbalance between the body’s oxidation system and its antioxidant system. In this condition, the body generates excessive amounts of reactive oxygen species (ROS) and their metabolic products, which accumulate within cells and exert toxic effects, resulting in cell death or apoptosis [16]. Under normal physiological conditions, the body possesses multiple enzyme and non-enzyme antioxidant systems capable of eliminating small quantities of ROS and its metabolites that are generated within cells. The former encompasses superoxide dismutase (SOD), glutathione peroxidase (GSH-Px), and catalase (CAT); the latter primarily consists of antioxidants such as glutathione, tocopherol/vitamin E, and vitamin C [17]. A small quantity of ROS is a normal product of cell metabolism and functions as a secondary messenger in cell signal transduction [18]. Nevertheless, when ROS accumulates in large amounts, it may induce persistent damage to cells, giving rise to impaired cardiac contractility, myocardial hypertrophy, and left ventricular remodeling, and thereby facilitating the progression of HFpEF [19] (Fig. 1).

## 4. The Origins of ROS in HFpEF

Nicotinamide adenine dinucleotide phosphate (NADPH) oxidase serves as the principal source of ROS in the cardiovascular system. There are more than 7 members of the NADPH oxidase (Nox) family [20], and Nox2 and Nox4 have the highest concentrations in the heart, constituting the main sources of oxidative stress [21]. Upon activation of Nox2, it induces the binding of NADPH to the C-terminus of the cell, and electrons are transferred from NADPH to oxygen on the opposite side of the membrane, thereby generating ROS [22]. Nox4 is regarded as constitutively active, and its expression level essentially determines its activity as well as the quantity of hydrogen peroxide (H_2_O_2_) produced within the cell [23]. Kuroda *et al*. [24] discovered in their genetically modified mouse model deficient in Nox4, that Nox4 might be the principal source of nicotinamide adenine dinucleotide (phosphate) (NAD(P)H)-dependent ROS production and engages in mitochondrial oxidative stress by regulating the facilitation of superoxide generation with NADPH as an electron carrier. Nitric oxide synthase (NOS), employing L-arginine as a substrate, catalyzes the synthesis of NO. NO functions as a vasodilator for endothelial cells and regulates the contraction and dilation of vascular smooth muscle cells via the NO-soluble guanylyl cyclase (sGC)-cGMP-protein kinase A/G (PKA/G) signaling pathway [25]. NOS encompasses three isoforms of enzymes: eNOS, neuronal nitric oxide synthase (nNOS), and iNOS [26]. Xanthine oxidoreductase (XOR), a molybdenum enzyme, participates in the generation of ROS [27]. This enzyme is extensively distributed in mammalian tissues and is typically a key enzyme in purine catabolism. It is capable of catalyzing the oxidation of diverse substrates and transferring electrons to molecular oxygen, thereby generating ROS [28].

**Fig. 2.**
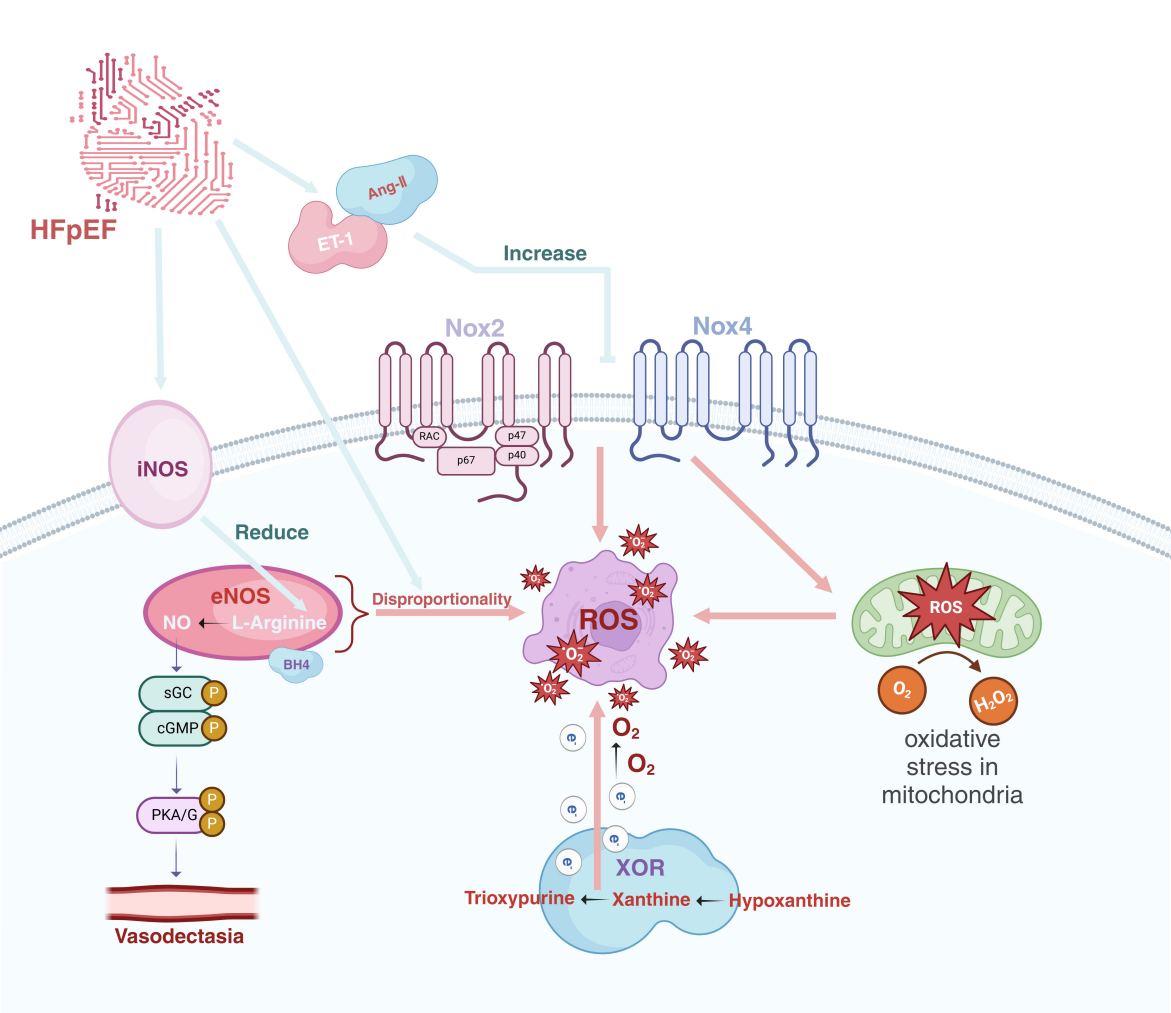
**The origins of ROS in heart failure with preserved ejection fraction**. ET-1, endothelin-1; Ang-Ⅱ, angiotensin-Ⅱ; iNOS, inducible nitric oxide synthase; eNOS, endothelial nitric oxide synthase; sGC, soluble guanylyl cyclase; cGMP, cyclic guanosine monophosphate; PKA/G, protein kinase A/G; Nox2, NADPH oxidase 2; Nox4, NADPH oxidase 4; XOR, xanthine oxidoreductase; ROS, reactive oxygen species; HFpEF, heart failure with preserved ejection fraction; BH4, tetrahydrobipterin; NADPH, nicotinamide adenine dinucleotide phosphate; H_2_O_2_, hydrogen peroxide. Created in BioRender.com.

In HFpEF, the activity of the RAAS system is increased, and multiple stimulating factors such as angiotensin II (Ang-Ⅱ) and endothelin-1 stimulate the cells, resulting in a significant increase in NADPH oxidase activity and further facilitating the generation of ROS [29, 30]. Furthermore, when HFpEF is present, an imbalance in the proportions of NOS might give rise to the dissociation of eNOS from the L-arginine catalytic reaction, culminating in the production of superoxide anions rather than NO products [31]. When superoxide anions react with NO to form peroxynitrite, it further enhances the generation of ROS [17, 32]. Research studies have found that iNOS expression is not detectable in normal cardiomyocytes; however, its expression is augmented in patients with HF [33]. iNOS participates in the oxidative stress process, and the sustained expression of iNOS leads to a reduction in L-arginine concentration, thereby diminishing NO production [34]. Recent data have also demonstrated that nNOS-derived NO assumes an important role in the pathophysiology of adverse cardiac remodeling [35]. Concurrently, in HFpEF, secondary ischemia and hypoxia give rise to augmented adenosine triphosphate (ATP) degradation in cardiomyocytes, resulting in elevated concentrations of the substrates xanthine and hypoxanthine, which leads to an increased production of hydrogen peroxide, NADH, and superoxide [36]. The increase in Ang-Ⅱ concentration indirectly potentiates the activity of xanthine oxidase (XO), ultimately culminating in an increased generation of ROS [37]. Landmesser *et al*. [38] discovered that the activity of extracellular SOD bound to the endothelium was significantly decreased, whereas the activity of extracellular XO was significantly augmented in HF patients in comparison with the control group. These findings imply that the increase in XO activity is intimately associated with oxidative stress in the vasculature of chronic heart failure (CHF) patients [38] (Fig. 2).

## 5. The Mechanism Underlying HFpEF Caused by Excessive ROS

Extensive research has been conducted on the mechanism through which ROS causes HFpEF. Study has demonstrated that the elevation of ROS has a significant influence on the electrophysiological activity of cardiomyocytes [39]. ROS reverses the Na^+^/Ca^2+^ exchanger (NCX), resulting in an increase in Ca^2+^ influx and Na^+^ efflux, as well as augmenting the activity of L-type calcium channels [6]. The abnormal activation of calcium channels can give rise to abnormal membrane potentials and further exacerbate mitochondrial ROS production, thereby further deteriorating HFpEF [7]. Calcium overload is capable of inducing mitochondrial rupture, decoupling of oxidative phosphorylation, release of pro-apoptotic factors, causing mitochondrial damage, exerting toxic effects on cardiomyocytes, and further contributing to the occurrence of HFpEF [40]. In recent years, investigations into the role of ferroptosis in oxidative stress have drawn widespread attention. HFpEF might be closely associated with the oxidative stress-induced upregulation of ferroptosis, a novel type of programmed cell death that differs from the apoptotic form [41]. Ferroptosis possesses distinctive morphologic and biochemical features, primarily manifested as excessive iron and lipid peroxidation [42]. Morphologically, cells exhibit necrotic-like alterations, encompassing the loss of plasma membrane integrity, cytoplasmic swelling, and swelling of cytoplasmic organelles. At the ultrastructural level, it is typically characterized by abnormal mitochondria, such as mitochondrial atrophy, increased membrane density, and decreased or absent cristae. The occurrence of ferroptosis is mainly associated with iron overload, lipid peroxidation (LPO), and glutathione (GSH) metabolism [43].

## 6. The Regulation of Ferroptosis-Related Mechanisms by Oxidative Stress

Ferroptosis is engendered by iron dependence and ROS-induced LPO [44]. The LPO triggered by the action of ROS on membrane polyunsaturated fatty acids (PUFAs) constitutes the direct cause of ferroptosis [45, 46]. Oxygen free radicals within ROS react with lipids to yield malondialdehyde (MDA), a highly toxic lipid peroxidation product that can further intensify oxidative stress and cellular damage [47, 48]. Glutathione peroxidase 4 (GPX4) and the cysteine-glutamate antiporter protein (SLC7A11) serve as core indicators of the ferroptosis pathway, and their downregulation gives rise to ferroptosis. Research indicates that knockdown of GPX4 and SLC7A11 in vascular smooth muscle cells (VSMC) can induce ferroptosis; however, the use of a novel ferroptosis inhibitor, SP2509, can nearly completely ameliorate ferroptosis caused by the knockout of GPX4 and other antioxidant systems [49].

LPO is intimately associated with GPX4 and SLC7A11. Wang *et al*. [50] discovered that LPO generated by ROS can lead to significant decreases in the transcription levels of GPX4 and SLC7A11, thereby inducing the occurrence of ferroptosis. Similarly, Dieterich *et al*. [51] discovered that in end-stage failing hearts caused by dilated (DCM) or ischemic (ICM) cardiomyopathy, the mRNA levels of various antioxidant enzymes changed. After conducting a study on these changes, they found that the increased oxidative stress in human end-stage heart failure may compensate by specifically upregulating *CAT* gene expression, without influencing the expressions of the *SOD* and *GPX* genes. This indicates that excessive ROS-induced LPO may deplete antioxidants, including GPX4, resulting in the accumulation of lipid peroxidation products and further triggering ferroptosis. Consequently, the upregulation of oxidative stress-related mechanisms for ferroptosis might play a crucial role in the emergence and deterioration of various diseases.

## 7. The Mechanisms and Clinical Applications of HFpEF Induced by Oxidative Stress via the Ferroptosis Pathway

Iron-dependent ferroptosis has been identified in failing heart cells, featuring characteristic mitochondrial structures such as mitochondrial contraction and an increased density of the mitochondrial membrane, which indicates that myocardial damage and the progression of HFpEF might be accompanied by ferroptosis. Studies have demonstrated that the inhibition of GPX4 activity or the reduction of GSH synthesis can aggravate myocardial fibrosis and compromise cardiac function [45, 52]. Cardiac fibrosis plays a vital role in the development of diastolic dysfunction and gives rise to HFpEF [53]. Geniposide activates the RNA-binding protein G-rich sequence factor 1 (Grsf1)/GPX4 axis to suppress oxidative stress and ferroptosis, resulting in protection against myocardial injury. Hence, ferroptosis might act as a bridge between oxidative stress and HFpEF [54].

### 7.1 The Mechanism of HFpEF Induced by Macrophage Iron Overload

Under physiological conditions, Fe^3+^ binds to transferrin and gains entry into cells via transferrin receptor 1 (TFR1), subsequently being reduced to Fe^2+^ and released into an unstable iron pool, and ultimately being excreted from cells through ferroportin 1 (FPN1). The process of iron absorption, utilization, and excretion constitutes a dynamic equilibrium. Macrophages within cardiac tissue assume an important role in iron homeostasis [55]. During HF, cardiomyocytes undergo damage, releasing heme proteins that augment the iron source, thereby resulting in iron overload of macrophages. Iron overload facilitates macrophage polarization into the M1 phenotype via the ROS/acetyl-p53 pathway, a process concomitant with the generation of a considerable amount of ROS [56]. ROS acts as a crucial signaling molecule that steers more macrophages to differentiate into the pro-inflammatory M1 phenotype rather than the M2 phenotype [57, 58]. M1 macrophages possess the capacity to secrete high levels of pro-inflammatory cytokines, which can give rise to chronic inflammation in the heart [59]. Excessive ROS generation induces lipid peroxidation of the membrane, adversely affects the activity of antioxidant enzymes, further provokes iron-dependent cell death in cardiomyocytes, induces cardiac dysfunction, and participates in the development and progression of HFpEF [60].

### 7.2 The Mechanism of HFpEF Resulting from Endothelial Injury-Induced Ferroptosis

The vascular endothelium serves as an important regulatory barrier for vascular homeostasis, and various oxidative stress factors can induce endothelial cell dysfunction by augmenting the production of ROS. Approximately 40% of HFpEF patients present with peripheral endothelial dysfunction [61]. Research has demonstrated that iron overload is correlated with endothelial dysfunction [62]. Moreover, iron overload can produce copious amounts of ROS via the Fenton reaction, giving rise to oxidative stress and mitochondrial damage, resulting in a decrease in NO bioavailability, further increasing myocardial cell iron death, ultimately leading to myocardial fibrosis and dysfunction in relaxation or contraction, and inducing the deterioration of HFpEF [63].

### 7.3 The Mechanism of HFpEF Induced by Mitochondrial Damage and Iron-Dependent Ferroptosis in Cardiomyocytes

Under physiological conditions, a considerable number of mitochondria exist in cardiomyocytes, accounting for approximately 30% of the total cell volume. About 95% of the energy demands are fulfilled by the oxidative metabolism of mitochondria to generate ATP, which is of crucial significance for cardiac function [64]. Mitochondria constitute the principal cellular organelles for oxidative metabolism and energy generation, and they partake in oxidative stress within cardiomyocytes [65]. During the process of oxidative metabolism, ROS are produced, thereby exposing the mitochondria to damage from oxidative stress, and resulting in injury to the cardiomyocytes [66]. The mitochondria can regulate the induction of ferroptosis by modulating the cysteine deprivation process. The process of cysteine deprivation is also accompanied by the production of lipid ROS [67, 68]. Based on this research, it is likely that iron death also occurs during this process.

In the presence of HFpEF, the sympathetic-adrenal system becomes hyperactive, inducing the myocardial cells to generate copious amounts of ROS. This leads to the impairment of mitochondrial DNA and increased mitochondrial permeability, resulting in irreversible damage to the mitochondria. When the mitochondria are compromised, they can produce additional ROS through mechanisms such as abnormal respiratory chains or mitochondrial calcium overload, further exacerbating mitochondrial damage and exerting toxic effects on cardiomyocytes, thereby further facilitating the occurrence of HFpEF [40].

### 7.4 The Mechanism of HFpEF Induced by Myocardial Fibroblast-Mediated Ferroptosis

**Fig. 3.**
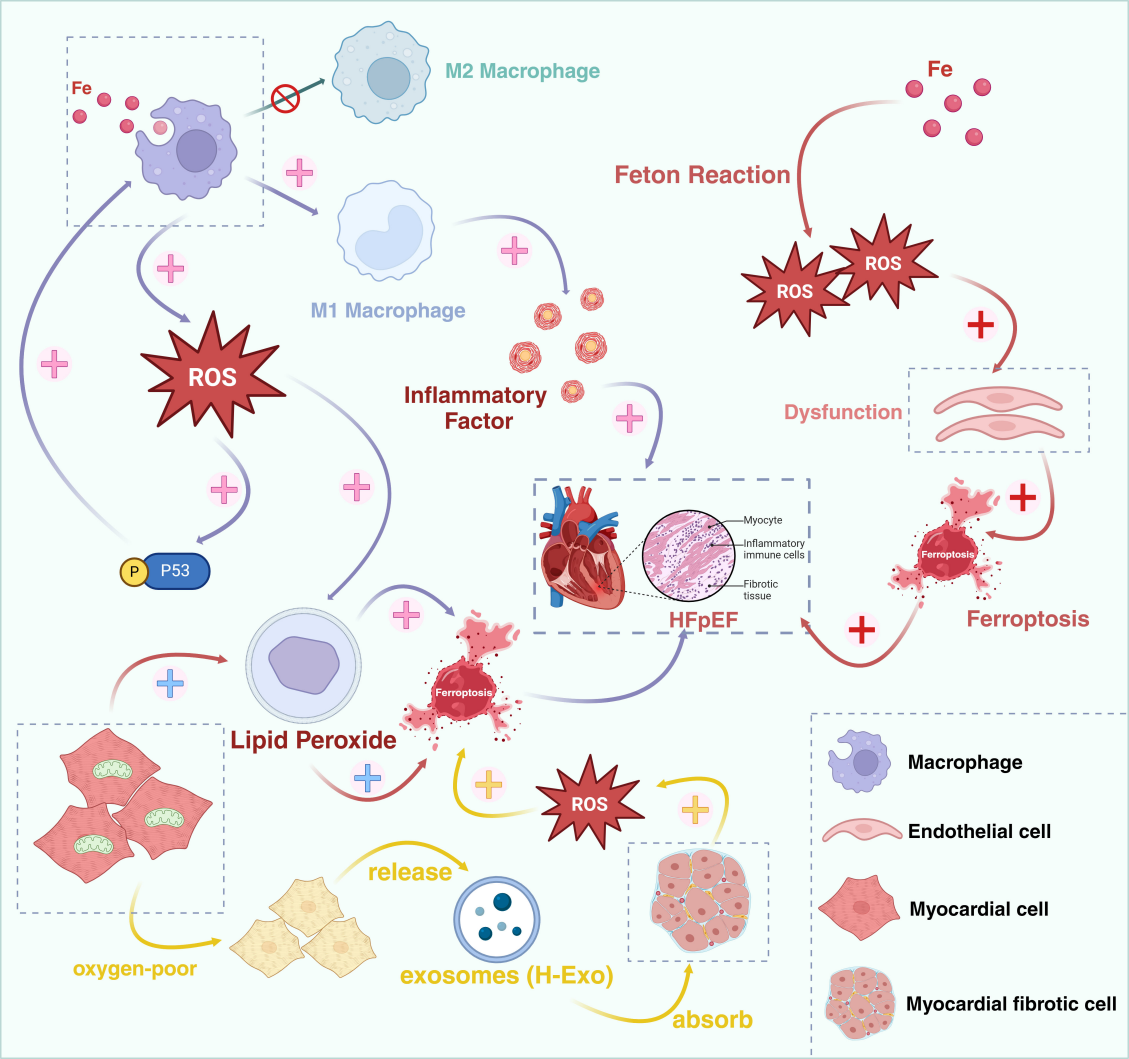
**Ferroptosis might act as a bridge between oxidative stress and HFpEF**. HFpEF, Heart failure with preserved ejection fraction; ROS, reactive oxygen species. Created in BioRender.com.

A study has demonstrated that the interactions between cardiomyocytes and fibroblasts regulate ferroptosis and fibrosis subsequent to cardiac injury [69]. Cardiac fibroblasts (CFs) play a crucial role in the repair process following cardiac injury by exerting paracrine effects and direct cell-to-cell interactions to safeguard cardiomyocytes and prevent ferroptosis. However, its abnormal activation may result in cardiac fibrosis. The hypoxic exosomes (H-Exo) released by hypoxic cardiomyocytes are abundant in miR-208a/b, and when H-Exo are absorbed by cardiac fibroblasts, they are capable of promoting the activation, migration, and ferroptosis of fibroblasts. H-Exo is capable of further augmenting the accumulation of ROS, MDA, and Fe^2+^, and suppressing the expression of GPX4 (a key regulator of ferroptosis) [69, 70]. Consequently, ferroptosis is associated with the activation of cardiac fibroblasts and is likely to facilitate the development of cardiac fibrosis, ultimately giving rise to HFpEF (Fig. 3).

### 7.5 The Ferroptosis-Related Pathways Induced by Oxidative Stress are also a Significant Target in the Prevention and Treatment of HFpEF

#### 7.5.1 The Oxidative Stress Mediated by Mitochondrial Iron Overload is Regulated by the Nrf2-ARE Signaling Pathway to Trigger Ferroptosis

Nuclear factor erythroid 2-related factor 2 (Nrf2) constitutes an essential transcription factor that assumes a central role in antioxidant defense. When cells are exposed to oxidative stress, Nrf2 translocates from the cytoplasm to the nucleus of the cell and binds to the antioxidant response element (ARE), thereby activating the expression of a series of antioxidant enzymes and other protective proteins, such as GPX4 [71]. Study has demonstrated that mitochondrial iron overload can give rise to oxidative stress in fish liver cells, resulting in the generation of excessive free radicals and the establishment of LPO, eventually triggering the occurrence of ferroptosis [72]. This process might be modulated by the Nrf2/ARE signaling pathway [72]. It has been reported that iron overload is capable of reducing the expression of Nrf2, which is in line with the experimental results of Jin *et al*. [73] demonstrating that ROS accumulation inhibited the expression of Nrf2 and facilitated its degradation in HepG2 cells. Previous studies have manifested that GPX4 and SLC7A11 are the transcriptional targets of Nrf2 [74, 75], and Nrf2 mitigates the occurrence of ferroptosis by directly binding to the promoters of GPX4 and SLC7A11 and activating them. Furthermore, the Nrf2 signaling pathway is capable of promoting the expression of VEGF, facilitating angiogenesis and tissue repair [76], resulting in a reduction in peripheral vascular resistance, which is of paramount importance for preventing the occurrence of HFpEF. Consequently, oxidative stress mediated by iron overload might induce ferroptosis through downregulating the Nrf2-ARE signaling pathway, giving rise to HFpEF.

#### 7.5.2 TLR4-Nox4 Signaling Pathway Induces HFpEF Induced by Ferroptosis

Nox4 constitutes one of the principal sources of ROS and participates in multiple pathways during the formation of fibrous tissue. It mediates the transition of fibroblasts to an active state and augments their capacity for collagen synthesis and secretion [77]. The high expression of Nox4 can additionally promote the formation of interstitial fibrotic tissue, thereby increasing ventricular wall fibrosis resulting in functional impairment leading to HFpEF [78]. The toll-like receptor 4 (TLR4)-Nox4 signaling pathway might be implicated in the process of HFpEF. Studies have found that the TLR4-Nox4 signaling pathway is augmented in HF and that the knockdown of either TLR4 or Nox4 concurrently reduces ferroptosis in HF [78, 79]. Moreover, the total extract of Abelmoschus manihot can efficaciously alleviate iron oxidative stress-induced ferroptosis in cardiomyocytes by downregulating Nox4, reducing ROS generation, and upregulating GPX4 and SLC7A11 [80]. Consequently, ferroptosis might serve as a downstream event of oxidative stress, initiated by the activation of the TLR4-Nox4 signaling pathway, resulting in a decrease in GPX4 and an increase in ferroptosis levels, which may be involved in the development of HFpEF.

### 7.6 Potential Clinical Utilization and Prevention

It has been demonstrated that in the absence of GSH synthesis, homocysteine (Hcy) can serve as a substrate to inhibit ferroptosis, and the inhibitory effect on ferroptosis mainly relies on the activity of GPX4 [81]. Nevertheless, Hcy can additionally promote LPO and intensify oxidative stress via the cysteine/glutamate antiporter (systemXc-)/GPX4 axis, significantly down-regulating the protein expression of GPX4 and inducing ferroptosis [82], which might be a side effect resulting from high levels of homocysteine. In animal experiments, Hcy has been employed to induce myocardial fibrosis and give rise to the occurrence of HFpEF [83]. It is evident that the increase of Hcy can give rise to oxidative stress and induce the occurrence and development of ferroptosis, ultimately resulting in HFpEF.

Clinical data has found that among patients with HFpEF, those with high Hcy levels have a significantly higher rate of severe heart failure (New York Heart Association (NYHA) IV grade) and incidence of cardiovascular adverse events in comparison with those with normal Hcy levels [84]. Hcy is a non-protein amino acid thathas been found to be a risk factor for atherosclerotic vascular diseases and arterial ischemic events. An elevated Hcy level significantly enhances the risk of cardiovascular and cerebrovascular diseases, which are also correlated with an increased risk of HF [85]. Consequently, the monitoring of Hcy has significant importance for the prevention of ferroptosis, thereby preventing the occurrence of HFpEF and ameliorating cardiovascular adverse events in HFpEF patients.

## 8. Oxidative Stress and Ferroptosis Exert a Predominant Role in the Evolution of HFpEF Induced by Anti-Cancer Drugs

### 8.1 The Potential Mechanism of TKIs-Apatinib Causing HFpEF

In recent years, substantial progress has been achieved in the development of novel targeted anti-cancer drugs, prolonging the survival of cancer patients. Nevertheless, these new therapeutic approaches might induce severe cardiovascular adverse reactions, counterbalancing the benefits brought about by anti-cancer treatment. Hence, it is essential to carry out in-depth discussions regarding these therapies. TKIs primarily inhibit VEGF by diminishing the activity of the vascular endothelial growth factor receptor (VEGFR) tyrosine kinase [86, 87], and are currently employed in the treatment of colorectal cancer, breast cancer, and kidney cancer. However, they also give rise to hypertension, which is the most common side-effect and can further lead to arrhythmias, and heart failure [88]. The VEGF pathway plays a crucial role in angiogenesis. Nevertheless, blocking the VEGF pathway may result in endothelial dysfunction and related cardiovascular complications, such as the occurrence of HFpEF [89]. Apatinib (apatinib mesylate) is an anti-cancer drug independently developed by China and belongs to the TKIs class. It has been primarily utilized for the treatment of solid tumors, such as advanced gastric cancer or adenocarcinoma of the gastroesophageal junction [90, 91]. Research findings have indicated that ferroptosis is correlated with endothelial dysfunction within the VEGF pathway [92]. Oxidative stress plays a significant role in the cardiotoxicity induced by anti-cancer drugs [93]. Consequently, apatinib might be involved in the progression of HFpEF via oxidative stress and ferroptosis pathways, and the relevant mechanisms of its induction of HFpEF are as follows:

#### The Underlying Mechanisms of HFpEF Induced by the Ras homolog family member A (RhoA)/Rho-associated coiled-coil kinase (ROCK)/mixed lineage kinase 3 (MLK3) Signaling Pathway

Researches have demonstrated that MLK3 is regarded as being associated with diverse diseases and plays a crucial role in causing cardiac dysfunction [94, 95]. In the early phase of chronic heart failure, MLK3 predominantly mediates the inflammatory response via the nuclear factor-kappa B (NF-κB)/NOD-like receptor family, pyrin domain containing 3 (NLRP3) signaling pathway, inducing cell pyroptosis and further facilitating the occurrence of myocardial fibrosis. In the late phase of chronic heart failure, it mainly mediates the induction of myocardial fibrosis through the c-Jun N-terminal kinase (JNK)/p53 signaling pathway by regulating oxidative stress and ferroptosis [96]. Ferroptosis is a non-apoptotic form of cell death that is triggered by iron-dependent lipid peroxidation and has been implicated in the pathogenesis of several inflammation-related diseases [97, 98]. The augmented inflammation during the anti-tumor process might also give rise to cardiomyocyte damage via this pathway. The NLRP3 inflammasome may also participate in the occurrence and development of ferroptosis. Our team has previously demonstrated that Apatinib can lead to an increase in blood pressure in Wistar Kyoto (WKY) rats via the RhoA/ROCK signaling pathway [15]. It was also discovered that the RhoA/ROCK signaling pathway participates in the mechanism of hypertension resulting from apatinib treatment in mice in a gastric cancer model [99]. Concurrently, it was identified that the RhoA/ROCK signaling pathway was involved in cardiac damage induced by apatinib treatment for gastric cancer via the NF-κB. A close association also exists between MLK3 and the RhoA/ROCK signaling pathway.

MLK3 is a mitogen-activated protein kinase (MAPK) kinase kinase (MAP3K), which participates in regulating signaling pathways within cells [100]. RhoA constitutes a member of the Rho family of small G proteins. MLK3 promotes the activation of RhoA via its kinase activity, thereby influencing the activity of the RhoA/ROCK signaling pathway and regulating the structure and function of the cell cytoskeleton through the RhoA/ROCK signaling pathway [101, 102, 103]. It was discovered that MLK3, a downstream factor of the RhoA/ROCK signaling pathway, collaborates with RhoA in the process of cell migration and invasion [104]. In conclusion, both MLK3 and the RhoA/ROCK signaling pathway are involved in regulating the physiological and pathological processes of cells. Consequently, apatinib might induce oxidative stress via the RhoA/ROCK/MLK3 signaling pathway, resulting in the occurrence and deterioration of HFpEF by upregulating ferroptosis. However, a concern is whether the prevention of HFpEF caused by such drugs will have an impact on their anti-cancer efficacy. In fact, studies have demonstrated that RhoA/ROCK activation can facilitate the spread and invasion of tumors [105], while the blockade of ROCK can enhance the activation of dendritic cells and T cells, suppress tumor growth, promote cancer cell phagocytosis, and induce anti-cancer immunity [106]. ROCK inhibitors have been verified to significantly prevent tumor growth, invasion, and metastasis [107]. Consequently, the research on the relationship between MLK3 and the RhoA/ROCK signaling pathway is vital to our understanding of the regulatory mechanisms of cell signaling networks and offers new targets and strategies for the treatment of HFpEF induced by novel targeted anti-cancer drugs.

### 8.2 Anthracycline-Based Anti-Cancer Drugs and HFpEF 

Anthracyclines constitute a class of chemotherapy drugs, encompassing doxorubicin, epirubicin, and daunorubicin, and are widely employed for the treatment of hematological malignancies and solid tumors [88, 108, 109]. Doxorubicin (DOX) is one of the most efficacious and extensively utilized anti-cancer drugs; however, it also gives rise to notable side effects, among which cardiotoxicity is the most common and severe [110]. In the initial stages of treatment, patients might encounter mild arrhythmias, cardiac dysfunction, and cardiac hypertrophy [111, 112]. In animal experiments, it was demonstrated that the hearts of mice treated with DOX for 6 weeks exhibited myocardial fibrosis. In comparison with the DOX group, the degree of myocardial fibrosis in the DOX + empagliflozin (EMPA) group was decreased by 50% [113]. Consequently, in the initial stage of treatment, DOX might induce the occurrence of HFpEF. Nevertheless, because of DOX’s dose-dependent and cumulative cardiotoxicity, the risk of HF rises with the increase in the accumulated dose, ultimately resulting in total heart failure [114, 115]. The primary mechanism of DOX-induced cardiac injury encompasses mitochondrial dysfunction resulting in an augmentation of intracellular ROS and oxidative stress [116], the relevant pathways of cardiomyocyte death include autophagy and ferroptosis [117]. Therefore, we might infer that DOX can induce HFpEF via the upregulation of ferroptosis through oxidative stress in the early stage of treatment.

## 9. Potential Therapeutic Approaches

Research has demonstrated that upon obtaining HFpEF chip data from the National Center for Biotechnology Information (NCBI) database and employing Gene Ontology (GO) and Kyoto Encyclopedia of Genes and Genomes (KEGG) enrichment analysis, significant enrichment of differentially expressed genes (DEGs) in iron-dependent pathways was noted in HFpEF [9]. Moreover, we have previously discovered that apatinib can induce hypertension, which can subsequently result in left ventricular hypertrophy and trigger HFpEF. This process also involves the occurrence of oxidative stress [99]. Hence, for HFpEF patients with comorbidities such as hypertension, diabetes, or chronic kidney disease, the underlying mechanisms are more intricate, but might encompass oxidative stress and ferroptosis. We can employ some antioxidant drugs. Among them, the novel antihypertensive drug endothelin-1 antagonist aprocitentan has been demonstrated to alleviate mitochondrial oxidative stress in human cardiac fibroblasts [118]. Its mechanism involves inhibiting the proliferation of human cardiac fibroblasts (HCF) induced by transforming growth factor-β (TGF-β) and reducing the generation of mitochondrial ROS. sodium-glucose cotransporter type 2 (SGLT-2) inhibitors which lower blood sugar and enhance kidney function, and confer benefits to the cardiovascular system by reducing oxidative stress [119]. For patients in whom HFpEF was induced by anti-tumor drugs, we can also employ a combined modality with Chinese herbal medicine. Flavonoids, a category of natural secondary metabolites, are mostly found in plants and have recently gained increasing significance as anti-cancer drugs [120]. Research has indicated that flavonoids can scavenge free radicals and alleviate oxidative stress [121], thereby offering protection against cardiotoxicity. They exert their antioxidant and stress-mitigating effects by modulating the Nrf2/heme oxygenase-1 (HO-1) signaling pathway [122]. Furthermore, these compounds are capable of enhancing mitochondrial homeostasis, restraining excessive cytochrome C generation, and inhibiting cell apoptosis [88, 123]. Research has demonstrated that naringin, a flavonoid present in citrus peels, can suppress ferroptosis by regulating the Nrf2/System xc-/GPX4 antioxidant pathway and alleviating myocardial injury [124]. Turmeric possesses a diverse range of biological activities (namely antioxidant, anti-inflammatory, anti-cancer, and antibacterial) [125, 126, 127]. In recent years, numerous researchers have demonstrated that curcumin has the capacity to alleviate oxidative stress and related disorders [127, 128]. Turmeric extract is also capable of mitigating the cardiac injury triggered by ferroptosis in diabetic cardiomyopathy via the Nrf2 pathway [129]. Consequently, patients suffering from HFpEF need to undergo evaluation by a multidisciplinary team, to provide the most optimal and safest treatment to enhance the prognosis for this group of patients.

## 10. Conclusions and Prospects

Oxidative stress contributes to the development and advancement of HFpEF by upregulating the ferroptosis pathway. Consequently, the exploration and intervention of the ferroptosis mechanism present novel perspectives for the treatment of HFpEF. This article offers a theoretical foundation and guidance for the clinical treatment of HFpEF by investigating the regulatory mechanisms of oxidative stress and ferroptosis-related pathways as well as their research progress and applications. Nevertheless, the ferroptosis regulatory mechanism and signaling pathway are intricate and have not yet been fully clarified, and require further in-depth exploration to disclose the fine molecular mechanisms of ferroptosis and to provide more scientific evidence for targeting ferroptosis in HFpEF. Additionally, the cardiovascular events triggered by the targeted anti-cancer drug apatinib continue to be of major clinical concern, as endothelial dysfunction is regarded as one of the crucial factors in the occurrence of HFpEF. An increasing number of studies have indicated that apatinib might directly or indirectly induce HFpEF. The specific mechanisms involved in this process are reviewed in this article, which suggest that upregulating the ferroptosis pathway via oxidative stress may result in new breakthroughs in the treatment of HFpEF caused by targeted anti-cancer drugs.
